# The role of culture in effective HIV/AIDS communication by theatre in South Africa

**DOI:** 10.1080/17290376.2014.903809

**Published:** 2014-04-04

**Authors:** Chijioke Uwah

**Affiliations:** PhD in Applied Theatre, is a senior lecturer at the Department of English and Comparative Literature, University of Fort Hare, Alice, South Africa, Email: Chijiokeuwah@gmail.com

**Keywords:** theatre, culture, communities, effective communication, HIV/AIDS, le théâtre, la culture, les communautés, la communication efficace, le VIH/SIDA

## Abstract

The need to effectively communicate HIV/AIDS messages in South Africa, given the high prevalence of the pandemic, cannot be overemphasised. Communication scholars have long emphasised the need to recognise adherence to cultural norms of target communities as catalyst for effective HIV/AIDS communication. Unfortunately this call has not been totally heeded by the designers of HIV/AIDS communication instruments. In the case of theatre, research has shown that in South Africa, theatre groups have gone into communities with pre-packaged plays without due cognisance of the cultural norms and beliefs of the target population. This research was conducted in KwaZulu-Natal (the province with the highest prevalence rate of HIV/AIDS infection in South Africa). Using a qualitative research methodology this paper investigated the inclusion/non-inclusion of the cultural norms of the target population in the design of the dramatic performance by the theatre group in its HIV/AIDS campaigns. The findings indicate that while the group did try to incorporate aspects of the cultural norms of the target population, it did so at a level that failed to effectively communicate the HIV/AIDS message to its audiences. This paper therefore seeks to show through empirical evidence that the non-inclusion of cultural norms and values of the target population has acted as a stumbling block in the effective communication of HIV/AIDS messages by theatre groups in the country.

## Résumé

La nécessité de communiquer efficacement des messages du VIH/SIDA en l'Afrique du Sud, étant donné la forte prévalence de la pandémie, ne peut pas être surestimée. Les savants experts de la communication ont longtemps insisté sur la nécessité de reconnaître l'adhésion aux normes culturelles des communautés cibles en tant que catalyseur pour une communication efficace au VIH/SID. Malheureusement, cet appel n'a pas été totalement tenu compte par les concepteurs d'instruments de communication sur le VIH/SIDA. Dans le cas du théâtre, la recherche a montré qu'en l'Afrique du Sud, des groupes de théâtre ont disparu dans les communautés avec des jeux pré-emballés sans dûment compte des normes et des croyances de la population cible culturelles. Cette recherche a été menée dans à KwaZulu-Natal (la province avec un taux de séroprévalence du VIH/SIDA en l'Afrique du Sud). Grâce à une méthodologie de recherche qualitative ce document étudié l'inclusion/non-inclusion des normes culturelles de la population cible dans la conception de la performance spectaculaire par le groupe de théâtre dans ses campagnes contre le VIH/SIDA. Les résultats indiquent que, bien que le groupe a essayé d'intégrer les aspects des normes culturelles de la population cible, il l'a fait à un niveau le pas à communiquer efficacement le message du VIH/SIDA à ses publics. Ce document vise donc à montrer par des preuves empirique, que la non-inclusion de normes et valeurs culturelles de la population cible a agi comme un horlage d'achoppement dans la communication efficace des messages sur le VIH/SIDA par des groupes de théâtre dans le pays.

## Introduction

The term “culture” traces its root back to German Romanticism and Herder's idea of the *Volksgeist* (the spirit of a people), which was adopted for anthropological use by Adolf Bastian. From Bastian the term diffused (via Edward B. Tylor) into British anthropology (where it received great prominence), and (via Franz Boas) into American anthropology (where it came to define the very subject matter of anthropology). Nevertheless, in one of the many paradoxical turns of the history of anthropology, it is Tylor's definition that is most often cited as classical (http://www.anthrobase.com/Browse/Thm/C/culture.htm).

According to Tylor, the term “culture” was used to denote the totality of the humanly created world, from material culture and cultivated landscapes, via social institutions (political, religious, economic among others), to knowledge and meaning. Tylor defines culture as “the complex whole which includes knowledge, belief, art, morals, law, custom, and any other capabilities and habits acquired by man as a member of society” (Tylor 1958 [1871]:1) (Mhtml: file://E\Culture – AnthroBase – Dictionary of Anthropology A searchable database of anthropology). On the other hand, UNESCO defines culture as a set of distinctive spiritual, material, intellectual and emotional features of a society or a social group and it encompasses in addition to art and literature, lifestyles, ways of living together, value systems, traditions and beliefs (UNESCO 2000).

Interestingly, New Partnership for Africa's Development (NEPAD) also defines culture in a very precise and brief manner as “the totality of life of a particular group of people” (http://culturalrights.net/imagenes/drets_culturals59.jpg). NEPAD sees culture as a capital that would enable Africa affirm and preserve her cultural identity, values and institutions, tangible and intangible heritage and make them the core for Africa's development. If culture is the totality of life of a particular group of people, it stands to reason that in Africa, culture and health are intertwined.

Mazrui (1986:239) defines culture as “a system of interrelated values active enough to influence and condition perception, judgement, communication and behaviour in a given society”. Hahn (1995) emphasises the role of culture and society in relation to sickness and healing, and highlights the use of language in the understanding of illness concepts. Lupton (1994:12) postulates that “the practice of medicine is a cultural production, particularly with respect to the focus on the body rather than the contexts that define and shape the body”. Airhihenbuwa and Webster (2004) are of the opinion that regardless of the disciplinary basis in which the definition is presented, it is generally accepted that culture is the foundation on which health behaviour in general, and HIV/AIDS in particular, is expressed and through which health must be defined and understood.

According to them, “the realisation of cultural centrality to health has resulted from the need to question and examine critically, the assumption inherent in western-based conventional theories and model, which postulate that health behaviour is a-cultural”. It is not surprising, therefore, that health issues are defined in terms of culture and therefore it is impossible to separate the two.

## Theoretical framework

Research has indicated that health communication in HIV/AIDs in Africa cannot be effective without due emphasis on cultural norms and values (Airhihenbuwa & Webster 2004). Accordingly therefore, Somma and Bodiang (2003:10) argue that “throughout the years of prevention efforts, it has become increasingly clear that conventional public health awareness campaigns are largely unsuccessful at eliciting behaviour change where sexuality is concerned. In part this is because behaviour patterns are not influenced by individual decisions but also deeply embedded within the cultural norms that are inherited”.

It is important to note that culture does not exist independently of individuals. On one hand, it is by means of their own culture that social factors interpret and shape their lives and environment and, on the other hand, culture is a dynamic construct which can also be subject to change.

With HIV/AIDS reaching such an alarming rate in South Africa, the need for theatre to adopt culture-sensitive models/theories in its health communication drive is vital. Airhihenbuwa (2007:37) believes that:

much of the theorising about Africa has been done through principles that have been framed in non-African cultural, geo-political and historical spaces. Indeed the language of the universality that assumes that theoretical truths, deployed through the prism of psychologism and anthropologism are universal truths. This has led to the design and implementation of interventions designed to offer solutions at the exclusions of identity in whose contexts problems and solutions are best understood.

Critics believe that many health communication projects have failed principally because the facilitators of those projects possessed very little understanding of the cultural norms of their target communities, and this contributes negatively to the conscientisation process. Green (1999:16) is of the opinion that “if there are to be effective interventions, it is more important than ever to understand how Africans (and others) understand contagious diseases”.

In central Mozambique, as well as Bangladesh for instance, Green says, several kinds of children diarrhoea and/or dehydration are believed to be caused by contact with polluting essences. One source of pollution that may appear mystical is unfaithful behaviour on the part of the parent: if a mother or father commits adultery, he/she acquires a contaminating essence that makes the child sick. The immediate cause is physical contact with the child. (Green 1999:13–14)

Green believes that to ignore a basic cultural belief such as the one mentioned above offends members of such communities. When practitioners design health communication programmes that offend the cultural sensibilities of their target audience, there is every possibility that they will achieve zero success in their quest to change the behaviour of such target audience (1999).

Dudley (1993) used the term “abandoned house” to describe villagers’ resistance to an imposed idea. According to him, when villagers feel offended about an imposed idea, they politely participate while the facilitators are around but abandon the idea as soon as the facilitators leave. Airhihenbuwa and Webster (2004:31) contend that “the fact that there are challenging health issues and seemingly intractable problems in African countries that deserve immediate and long term solutions is without debate. What has been, is, and remains an issue, he further contends, is on whose experience and in whose tradition of knowledge production should the solutions to these issues and problems be anchored”. In many parts of the third world, developmental projects are initiated without due input from the target population. In most HIV/AIDS campaigns, the initiative usually comes from the middle class, often based in universities or university qualified, and lack participation by target population. In most instances, these campaigners often go to these communities with “superior knowledge” regarding healthy behaviour by the communities. Most theatre groups in South Africa often campaign in communities armed with pre-packaged plays which are performed before a large audience of rural or urban rural communities referred to as *townships* and *informal settlements.* Many of these plays are prescriptive and reflect the groups’ opinion of what safe sex behaviour is. Neither the cultural dynamics nor the socio-economic realities of these people are seriously reflected in these productions. Consequently, not much is achieved in terms of behaviour change as far as HIV/AIDS is concerned, despite theatre's insistence of success in their glossy brochures prepared for the benefit of donor agencies. The need to incorporate the cultural norms of target communities for effective HIV/AIDS communication can therefore not be over-emphasised.

Gould (2007:3) states that

As people are entangled in different cultural webs, to be affected by a message, people have to hear it in a way that has cultural significance for them and which connects with their experience of life. Culture has a potential to connect with people and affect them on many different levels. However, communication programmes have tended to focus largely on one level of behaviour change.

A cultural approach offers a chance to improve the effectiveness of global HIV/AIDS strategy and rebuild the trust of communities through more sensitive modes of engagement. In so far as a cultural approach allows prevention and care methods to come from within the culture, it “maintains socio-cultural ownership and credibility” (Somma & Bodiang 2003:19). Local community-based approaches driven by “community work” and “social activism” will remain the most important means of influencing people (Lynn 2004).

“It is therefore crucial”, says Airhihenbuwa and Webster (2004), that in the design of health communication programmes on the continent,

the African identity of target audience occupy a central position. There is also a need to deconstruct conventional assumptions and theories that have been used to frame public health issues and solutions in the continent. Equally important is the need to insist that culture be central to how issues of health and behaviour are formulated.

Culture has been positioned in the foregoing as a major factor in the various ways that HIV/AIDS has impacted on the African population. It is important, therefore, that any health intervention in Africa should have culture as a priority factor in the design of its health communication programme.

Somma and Bodiang (2003:1) agree and argue that:

culture is one of many factors influencing human behaviour; it is a determinant of socially accepted behaviour, value systems, beliefs, and practical knowledge. Means of expression or communication, such as music, dance, theatre, and art, are those creative aspects of culture that we often define narrowly as culture itself. However, culture in the broader sense, includes also traditions and local practices, taboos, religious affiliations, gender roles, marriage and kinship patterns and so forth. Therefore culture is deeply rooted in all aspects of a society, including local perceptions of health and illness and health seeking behaviours.

Broadly speaking, like fish that lives in water, a human being is a creature of culture and life without it is worthless. Airhihenbuwa and Webster (2004:5) concur by comprehensively stating that:

culture has been shown to have both positive and negative influences on health behaviour. Indeed, culture is often shown to be a factor in the various ways that HIV/AIDS has impacted on the African population. It is important to note that culture does not exist independently of individuals. On one hand it is by means of their own culture that social actors interpret and shape their life and environment, and on the other hand, culture is a dynamic construct which can also be subject to change.

Therefore, “it is important to gain an understanding of the social and cultural contexts of people's lives and to identify needs within, and in terms of, such contexts” (Heggenhougen 1991:21). In that sense, a cultural approach in health issue utilises culture as a lens through which one can gain a greater understanding of individual and collective health behaviours, and a means to formulate prevention programmes within a specific cultural context.

Based on this, Heggehougen (1991:21) defines culture as an entity that “provides people with a way of perceiving the world at large and with ways of coming to terms with the problems they face: [including] attitudes about the body and ways in which a person should be treated when ill”. Bringing cultural approach into HIV/AIDS work allows for prevention efforts not to rely solely on the import of foreign and biomedical concepts as means of prevention, but also to utilise indigenous knowledge systems for sustainable and appropriate health programmes and prevention efforts. This is especially true in Africa where decades of health interventions have failed to achieve the desired change in the sexual behaviour of most target communities. In the words of Somma and Bodiang (2003:4):

despite more than a decade of work in the field of HIV/AIDS prevention, global estimates of HIV infections now stand at a staggering 40million. The advent of the pandemic has forced public health professionals to reanalyse their priorities and methods. Throughout years of prevention efforts, it has become increasingly clear that conventional public health awareness campaigns are largely unsuccessful at eliciting behaviour change where sexuality is concerned. In part this is because behaviour patterns are not only influenced by individual decisions but abo deeply embedded within collective cultural norms that are inherited.

In response to the dangerous threat of HIV/AIDS, several large-scale HIV/AIDS communication campaigns have been undertaken. Unfortunately, these communication campaigns have proved unsuccessful in achieving the kind of behaviour change required to effectively address the pandemic (Parker 2006; Swanepoel 2005). One of the main reasons for this lack of success is the complexity of the behaviour these interventions are trying to change (Perloff 2001). Another reason is that the designers of these interventions are not in touch with the latest research developments in the field of health communication (Hoeken & Swannepoel 2008:2).

In a three-year project funded by the UNAIDS to develop a new direction for HIV/AIDS prevention in Africa, Asia, Latin America and the Carribbean, culture was one of the five key domains that was recommended to become central in HIV/AIDS prevention, care and support, particularly in Africa (Airhihenbuwa, Makinwa & Obregon 2000). Brody (1987) contends that one's cultural belief system influences one's social roles and relationships especially when one is ill. Conventional public health awareness campaigns seem to have failed in the past to elicit behaviour change because they were performed outside the social and cultural contexts of the target audience. In this case people may hear the message and understand them but fail to contextualise these messages within the realms of their culture (Somma & Bodiang 2003).

Van Dyk (2001:2) believes “that many Western-based AIDS education have failed dismally in Africa and they may only succeed if traditional African beliefs and customs are taken into account”. The need for cultural sensitivity therefore becomes crucial to the planning and execution of HIV/AIDS interventions. Resnicow, Soler, Braithwaite, Ahluwalia & Butler (2000:272) define cultural sensitivity as “the extent to which ethnic/cultural characteristics, experiences and beliefs of a priority population as well as relevant historical, environmental and social forces are incorporated in the design, delivery and evaluation of targeted health promotion materials and programmes”.

In order to accomplish cultural sensitivity of programmes, it has been suggested that interventionists have to consider both the explicit or surface cultural manifestations such as language, clothing, contexts and traditions, and implicit or deep manifestations of culture such as beliefs, values, norms and roles (Resnicow, Baranowski, Ahluwalia & Braithwaite 1999; Resnicow *et al.* 2000; Wilson & Miller 2003). A number of strategies have been advanced to accomplish cultural sensitivity (Kreuter & McClure 2004; Wilson & Miller 2003). The first is the presentational strategy which refers to peripheral evidential and linguistic strategies enhancing message receptivity and accessibility, for instance; it is about the use of native language and cultural sensitive scripts and contexts. The second strategy, known as socio-cultural strategy, refers to approaches used to enhance message salience by grounding the intervention content in the context, experience, beliefs and norms of the priority population. Finally the constituent strategy refers to the active participation of members of the target cultural group in the programme design process and employing the assistance of peer health educators.

## Context for the study: KwaZulu-Natal province

From the 28th of February to the 4th of March 2011, the researcher accompanied DramAide, a theatre group based in

**Table 1.** Sample questions for focus groups and individual interview.

What is your impression of the play you have just seen?
Tell me what aspects of your culture are represented or not represented in the play?
What do you think about the way the play presents the realities of HIV/AIDS in your community?
How well did the play highlight factors that contribute to HIV/AIDs in your community?
How do you feel about the group repeating this performance in your school?
What do you think of the idea of working with the group in writing the play?

KwaZulu-Natal, on tour of three high schools in villages and townships around Pietermaritzburg in an area called Sweet River. The schools visited were Siyanda High school, Ikusaselihle High school and Willowfontein High school. The group performed a play titled Scrutinize Your Behaviour to learners in the above-mentioned schools.

The policy of most schools is that the time for activities like this performance must not interfere with normal class times and so the plays were performed during the break period around 10 am. The play lasted a maximum of 30 minutes and post-performance discussions lasted 25 – 30 minutes, based on the arrangements I made with the respective school authorities. The individual interviews with the performers took place immediately after each performance for about 30–45 minutes. The focus group interviews took place thereafter with six learners per school and lasted for about 60 minutes. The individual interviews with the Life-Skills teachers followed and lasted for about 30 minutes.

The questions given in Table 1 were asked:

From the questions asked, the following themes and categories were derived. However, some of the themes and categories that form the basis for the analysis are derived from Resnicow *et al.*‘s (2000) definition of cultural sensitivity, which includes (1) Peripheral linguistic strategy that refers to language and culturally sensitive scripts and contexts, (2) Socio-cultural strategy which refers to context, experiences, values, beliefs and norms of priority population and (3) Constituent Strategy which refers to active participation of members of the cultural group of interest in the design of the play (Table 2).

## Synopsis of the play: scrutinise your behaviour

The play has three characters namely, Tshepo, MaZulu and Pinky. Pinky is the daughter to MaZulu. She is from a very poor family. The family survives on the meagre income Pinky's mother gets as a domestic worker. Pinky's mother is very strict and brought her up with the right values. Pinky lives by the family values her mother instilled in her. She carries herself with dignity and does not treat herself cheap. Because of her strong foundation in life, Pinky is able to resist peer pressure in her life despite her circumstances. She is outstandingly beautiful and attracts the attention of many men in her community.

**Table 2.** Themes and categories for focus groups and individual interviews.

Themes	Categories
Socio-cultural strategy	Cultural beliefs and norms
Peripheral linguistic strategy	Language Idioms Folklore Praise poetry Music and dance
Constituent strategy	Audience interactivity Audience participation
Sustainable intervention structures	Intervention frequency Building of structures to sustain gains of intervention
Perception of the play	HIV/AIDS education Dramatic presentation Non-representation of socio-economic realities

One day, she fell in love with a womaniser whose name is Tshepo. Despite the fact that she loved him very much she refused to have sexual intercourse with him, because he refused to do an HIV counselling and testing (HCT). He tried to persuade her to have sex but she refused. Tshepo became frustrated and reminded her of all the gifts he had given her. He then demanded that all the gifts he gave to her must be returned back to him. He went as far as demanding that Pinky vomit the food he bought for her from *KFC, Steers* and *Nandos.*

MaZulu is the mother of Pinky, a single parent whose husband had left her for another woman. He is not helpful at all and does not even support Pinky, his daughter. Since Pinky's father left her, Pinky has behaved well and kept herself, despite the fact that men were trying hard to get her attention. Her mother encourages her to focus on her studies and delay sex until she finishes her studies. She does not want to see her daughter hurt as well. She is very protective of her child and would not hesitate to fight anyone who interferes with her daughter. In this play she dealt strongly with Tshepo who wanted to have sex by force with her daughter.

Tshepo is a middle-aged man who is a womaniser and falls in love with all sorts of women from all walks of life. He specialises in dating rich professional women. He does not work because he comes from a rich family and drives a Mercedes Benz belonging to his mother. Sometimes he drives his father's Jaguar. He likes the fancy lifestyle. He constantly brags about sleeping with a minimum of 20 women every month. Unfortunately, he never uses protection everytime he has sex. Pinky demands that he take the HCT which he refuses and opted for Medical Male Circumcision (MMC).

However, Pinky explained to him that he will still need to take HCT since it is a prerequisite for the MMC. He opted for a condom which Pinky did not want to entertain since she is not ready for sex and has a friend who slept with her boyfriend and the condom burst and that frightened Pinky so much. Tshepo went to Pinky's home and tried to force Pinky to have sex with him and failed because her mother intervened at the right time but Pinky was already physically assaulted by Tshepo who was intent on having sex with her by force.

## Findings and discussions

### Socio-cultural/peripheral linguistic strategies (cultural norms, beliefs, language, etc.)

#### Learners

The learners from Siyanda High school were unanimous in their agreement that respect which is an integral part of Zulu culture did not feature much in this play. The group singled out the behaviour of the character of Tshepo who went to Pinky's house without showing respect to the parents. In Zulu culture, Tshepo should have paid lobola and gone there with presents as a mark of respect for the parents:

In the play, Tshepo's (main character) visit to Pinky's house was disrespectful in Zulu culture. In Zulu culture, young men are not allowed to visit their girlfriends at home unless lobola has been paid. (Respondent 3)

The group argued that the absence of a male father figure in Pinky's life is the main reason why Tshepo had the audacity to go to her house and try to take her by force. They concluded that the play should have included a male figure in Pinky's home typical of Zulu culture:

Where is Pinky's father or uncle? It is not nice to see Tshepo go to Pinky's house and beat her. Maybe he knows Pinky has no father. (Respondent 5)

Learners were not happy with the way Pinky responded to her mother's instructions. They felt she was disrespectful by refusing to sit down when her mother asked her to. They pointed out that the play did not show Tshepo's parents. The learners believe it is important to show the family's reaction to Tshepo's behaviour. More importantly, parents must be seen to be advising him against his behaviour.

The play did not include Tshepo's parents and what they think of his behaviour. (Respondent 4)

The learners from Ikusaselihle High school agreed that a key cultural element that was missing from the performance is the concept of reward and punishment. The group believes that the character of Pinky who has shown exemplary resilience by refusing to have sex with Tshepo in exchange for gifts should be rewarded. They felt that Pinky should have gone on to become successful in life in the play as reward for her moral strength so that the audience would see the reward for being good.

Pinky should have become a minister or lawyer for her good behaviour (Respondent 3)

The group also pointed out that the play failed to show Tshepo's other girlfriends who became sick with HIV as punishment for their waywardness as a warning to the audience. They were worried that for all his sexual carelessness, Tshepo was never really punished. The group agrees that the play should have included a scene in a clinic where Tshepo would be told he is HIV-positive:

Tshepo did not get punished. He did not get infected with HIV for all his carelessness. (Respondent 3)

They should show a clinic where Tshepo would be told that he is HIV positive. (Respondent 2)

#### Life-Skills teachers

While the Life-Skills teacher from Siyanda High School is of the opinion that certain cultural issues such as language were adequately addressed by the play, there are other cultural norms that were ignored by the designers of the play. One of them is respect, a central part of Zulu culture. Like the learners, she feels that the play should have avoided presenting a scene where the character of Tshepo went to Pinky's house to demand that she goes with him. This is disrespectful in Zulu culture where a young man is not allowed to visit his girlfriend at home unless he has paid lobola or bride price:

How can Tshepo go to Pinky's house to take her by force when he hasn't paid ilobola? That is not allowed.

She also commented about the absence of a stable family structure reminiscent of Zulu culture. The play did not show Tshepo's parents advising him about his wrong lifestyle. The play presented Pinky's mother as a single mother. The Life-Skills teacher is of the opinion that while single motherhood represents modern reality, a male figure in Pinky's household would have brought much needed stability:

The play did not show Tshepo's parents teaching him about HIV as in Zulu culture.

Single motherhood is not part of Zulu culture but shows modern life. They should have included Pinky's uncle.

The Life-Skills teacher from Ikusaselihle High School commented that certain aspects of culture were well represented in the performance. She said although single motherhood is not part of Zulu culture, it is part of modern reality. She believes the play also shows the manipulation of the rich by the poor in the portrayal of Tshepo's manipulation of Pinky's low economic status for his own gain. The only aspect of culture she felt should have been made part of the play is the inclusion of father figures in the lives of both Pinky and Tshepo in line with Zulu culture:

Pinky's father or uncle should have been in the play. Zulu's don't appreciate a home without a man in it (Respondent 1)

There was no father figure in Pinky's home when Tshepo went there to beat her. A man should be there to protect her. (Respondent 6)

Parental influence is a vital source of education and training in African culture. The Life-Skills teacher believes that young children watching the drama performance that was presented to them will internalise the message more if it is presented through the mouths of parents.

The Life-Skills teacher from Willowfontein High School said that aspects of Zulu culture were covered by the play; for instance, the character Pinky's insistence on not having sex with Tshepo before marriage is in line with Zulu culture's emphasis on virginity before marriage. Even Tshepo's physical abuse of Pinky is common among Zulu men who think it is their right to discipline a woman through physical abuse. In her opinion, the play covered all aspects of Zulu culture good and bad.

#### Performer-educators

The performer-educators insist that cultural norms and values were adequately covered in the play. They point to language as an example. When the researcher raised the issue of single motherhood as going against the Zulu and African culture, they said it was in keeping with modern realities.

Culture is very strong in this play. We are also trying to show modern happenings in our society.

### Constituent strategy (audience participation)

#### Learners

The focus group interviews revealed that the audience members were passive recipients of the groups’ “superior knowledge” on HIV/AIDS. The fact that they were not participants in the creation of the drama material became apparent in their responses to issues surrounding the cultural values as encapsulated in the play. The learners in all the schools visited in this province said they were not consulted during the writing of the play:

They never came to us for ideas (Respondent 3)

We are seeing the play for the first time. We don't know when it was written (Respondent 5)

They never came to our school (Respondent 1)

#### Life-Skills teachers

The Life-Skills teachers also confirmed that the group did not seek their input:

I didn't think about it but now that you raise the question, yes, we would have helped them with information about HIV and the behaviour of the community about it (Respondent 2)

If they had come to us we would have helped them understand our community better (Respondent 1)

The non-involvement of the target audiences and lack of their insight and contributions to the design of the play meant that there were gaps in the group's covering of cultural norms as well as creating a realistic performance that reflects all the realities of life of the target community. The fact that many of the schools visited were located in villages and township areas around Pietermaritzburg makes the issue of cultural inclusivity more relevant.

### Performer-educators

The performers agreed that they did not consult the audience in the design of the play. They argue that it is an expensive exercise:

We didn't go to the schools to get information. We were not told to do that (Respondent 1)

To visit all the schools and then come again to perform will be too expensive (Respondent 2)

### Sustainable intervention structures

#### Learners

The learners said they would like the group to come back and perform again:

The group should come back. (Respondent 3)

They must come again. Once is not enough. (Respondent 4)

#### Life-Skills teachers

The Life-Skills teachers in all the schools visited agreed that the once-off intervention by DramAide is not enough and efforts should be made to repeat the interventions as regularly as possible. They believe that this would help sustain the message in the minds of the learners. Alternatively, they would like the group to conduct training workshops on acting skills which would train them and the learners in educational theatre so that they would continue the process themselves:

The group should come regularly to our school (Respondent 2)

They should train us in drama so we can do it ourselves (Respondent3)

They make a useful contribution. They should come more often. (Respondent 1)

#### Performer-educators

The Performer-educators said it would be too expensive to conduct the intervention more than once in a school:

We operate within a budget. It would be too expensive to go round all the schools twice or more. (Respondent 1)

### Perception of the play

#### Learners

In a rather surprising twist during the focus group sessions, learners from Siyanda High school, Ikusaselihle high school as well as Willowfontein High school all agreed that the play Scrutinize Your Behaviour was very educational and that their knowledge of HIV/AIDS was enhanced by the play. They described the play as educational, good, enjoyable and informative:

I learned that I must get tested (Respondent 4)

The play taught me not to be afraid of getting tested (Respondent 6)

I learnt that it is not good for a girl to be with sugar daddies (Respondent 1)

I now know the meaning of HCT (Respondent 3)

While the learners are unanimous in their agreement that the play was educational, they expressed disappointment that the play did not deal with other issues that contribute to the spread of HIV/AIDS in their community. They are of the opinion that key issues such as rape and drug abuse were not addressed in the play:

There are problems in our communities such as rape, young boys smoking dagga and raping girls which did not appear in the play. These problems are for real in our community. (Respondent 1)

Many boys are raping their girlfriends and beating them (Respondent 4)

#### Life-Skills teachers

The Life-Skills teacher for Siyanda high school believes the play was educational and has assisted her by dramatising most of what she teaches in life orientation classes:

The play covered a lot of our lessons very well

The Life-Skills teacher for Ikusaselihle high school was full of praises for the theatre group. She says the play was very educational and the learners, judging by their reactions, were touched by the events in the play:

I could see some of the learners had experienced part of what Pinky (character in the play) was experiencing. The play has made a difference.

At Willowfontein High School, the Life-Skills teacher was equally impressed with the performance. She said she wished this dramatic performance would be a more permanent feature because the learners seem to resonate with the issues raised:

The issue of getting tested is a very big problem among youths. The play has dealt with it very well.

Despite the complimentary opinions of the Life-Skills teachers regarding the play, some of them felt that the play did not reflect certain realities of their society:

The Life-Skills teacher from Willowfontein High school said the play did not focus on some other key problems in society such as boys coming to school with guns and drugs.

Play is not realistic. It does not show problems such as drug abuse, boys smoking dagga, and drinking and raping girls.

The Life-Skills teacher from Ikusaselihle also commented on the play's inability to reflect society's realities and argued that there is no way Pinky's character would not have accepted the gifts from Tshepo if it was in real life. She said many girls here have sugar daddies and consent to having sex with them in exchange for gifts.

Pinky did not accept Tshepo's gift in the plays. Girls here accept gifts and give sex in return. (Respondent 1)

#### Performer-educators

The Performer-educators say they believe the play was very educational and the learners learnt from it and also enjoyed the performance.

The play was very inspiring. The kids learnt a lot. (Respondent 2)

The Performer-educators also said the play is a true reflection of society and its response to HIV/AIDS:

The play shows how people respond to the issue of testing. Many men are afraid to test (Respondent 2)

## Summary of responses

(Table 3)

## Conclusion

According to the table, a total of 42% being the total number of the respondents in both the focus group sessions and individual interviews identified aspects of the play that did not represent their cultural norms and values. A breakdown of the figures show that 66.67% of respondents in Siyanda High school and 50% of respondents in Ikusaselihle High school identified aspects of the play that failed to address their cultural values.

In total 11 out of 18 (learners) respondents in the three schools visited believed that key aspects of their culture were not represented in the drama performance. A total of two out of three Life-Skills teachers, which translates to 66.67% of Life-Skills teachers in the three schools, also criticised the play's non-inclusion of cultural values. Another area that received attention was the non-participatory nature of the group's intervention. A total of 21% of respondents from the three schools say the group left them out of the creative process of the intervention instrument.

The fact that only 42% of all the respondents in this province believe that cultural norms and values were not sufficiently reflected in the play means that DramAidE made considerable efforts to incorporate the cultural norms of the people in their performance. While this is a good sign of the group's seriousness in their HIV/AIDS campaign drive, there is still a long way to go before total success is achieved. However small the percentage may be, the views of this group of respondents cannot be ignored. The respondents’ opinions show that the group tried to compromise African norms and values by the inclusion of certain western values and in this way undermined respect which is central to African culture, the Zulus included. The Zulus regard respect as central to their culture. In their daily lives, Zulus place a high value on showing respect to others (Ndlovu, 2008, http://zuluroyals.com/zuluculture.htm). Any HIV/AIDS intervention in Africa that does not take cognisance of the cultural dynamics of any given community is bound to achieve limited success in its efforts (Uwah 2012). This research has revealed that in Africa, the best option for HIV/AIDS communication is to be rooted in the culture of the people concerned and not on health communication theories that have no relevance to the socio-economic and structural realities of the target population.

The combination of Western cultural infusion and African values in this play did not sit well with the audience who believe that the character of Tshepo was very disrespectful to the parent of the girl by going to her house and demanding to see her. The theatre group was trying to present the reality of modern culture in a community where people are still tied to their traditional culture and this did very little to communicate the message to the audience. It is also clear from the data that the group, which made the interventions in KwaZulu-Natal, relied on top-down communication which did not involve the target communities in the design of their material.

Due to this lapse, key aspects of their messages were lost on the audience, for instance, an important part of their message is “Get tested” which the audience failed to internalise because the main character, Tshepo, did not get tested throughout the play and no punishment was meted out to him for his sexual recklessness. Another key message was that young girls should not accept gifts from men in exchange for sex. This message was also lost because the respondents say the play did not reflect the realities of poverty in their communities. In real life, it is the girls who go after rich men for financial gratification in exchange for sex.

**Table 3.** The summary of the responses from KwaZulu-Natal province.

	Siyanda		Ikusasalihle		Willowfontein		Life Skills		Performers		Row (total)	Row (%)
	Count	Percentage	Count	Percentage	Count	Percentage	Count	Percentage	Count	Percentage		
Socio-cultural/peripheral	4	66.67	3	50.00	4	66.67	2	66.67	0	0.00	13	42
Constituent strategy	1	16.67	2	33.33	1	16.67	0	0.00	1	33.33	5	21
Sustainability	1	16.67	1	16.67	1	16.67	1	33.33	2	66.67	6	25
Total	6	100.00	6	100.00	6	100.00	3	100.00	3	100.00	24	100.00

A key element of African culture centres on the concept of reward and punishment. According to Green (1994), one of the reasons for the stigma in African societies is the fact that many people see HIV as punishment for one's wrongdoing. Among the *Tswana* people, the term *meila* refers to transgression against any of a number of taboos surrounding sexual relations and childbirth (Staugard 1985) and many sexually transmitted infections are attributed to *meila.* Even the Christian doctrine has a place for sin and punishment. During the group discussions, the fact came out very strongly as respondents felt the character of Tshepo was never punished for his sexual waywardness in the play. The respondents believe that as long as Tshepo's character escaped punishment the learners will regard the whole message as a joke.

The application of health behaviour theories needs to be addressed here. According to their annual reports the group under study bases its communication model on Bandura's theory of social learning. The theory of social learning is based on the premise that people learn through observing others behaviour, attitudes and outcome of those behaviours. According to Bandura (1977), most human behaviour is learned observationally through modelling; from observing others, one forms an idea of how behaviours are performed and on later occasions this coded information serves as a guide for action. This research has revealed that the application of this theory which is conceived in a western context on an African setting cannot work. In the first place, the western context in which this theory was conceived is very individualistic in its approach to life. This context operates on social cognition approaches which conceptualise the individual as a rational information processor whose behaviour is determined by a combination of psychological factors such as individual attitudes, personal action plans and perceived social norms (Campbell 2003). The African context on the other hand is very collectivist in its approach to life. Audiences respond to performances based on the dictates of their culture. Campbell (2003) believes that in Africa, local communities often form the contexts within which people negotiate their social and sexual lives and identities. These communities equally play a key role in enabling or restraining people from taking control over their health. While the theory is relevant and can be applied in most western contexts, it presents a problem when applied in a collective culture such as Africa where people pattern their behaviour on existing cultural norms. In a culture such that of the Zulus where respect is central, presenting characters that flout this cultural norm can only anger the audience instead of appealing to their sense of choice.

Again, the group did not take into account the structural realities of the given community. To create an ideal model in Pinky who chose to turn down material gifts from a boyfriend in a community where poverty is very high can only create distrust in the audience. It would be unwise to expect any young girl in the audience to emulate such ideal character when she does not know where her next meal is coming from. It must be emphasised that the government of South has in place a school feeding scheme in all primary and high schools in rural areas and townships across the country because most families are too poor to provide food for their school-going children.

Unfortunately, like most theatre interventions that do not adopt a full participatory strategy, this group's intervention did not address certain issues in the play that have a direct bearing on the levels of prevalence of HIV/AIDS in the community in a realistic manner. Many theatre interventions do not assess the economic condition of their target communities and reflect that in the play. Poverty as a key component of HIV prevalence is often overlooked. For this reason many plays always tend to misrepresent economic circumstance of the target community.

For instance, the reaction of a respondent to a particular scene in the play, where the female character refuses the sexual advances from her boyfriend in spite of the gift he had given her earlier in the play, shows that the scene does not reflect the reality on the ground. Thus, in trying to create a good role model in Pinky, based on the theory of social learning which underlines DramAidE's interventions, the group failed to understand the realities of poverty in that community where the decision-making power regarding sexual matters rests with the more affluent male benefactor.

Finally the author recommends that future HIV/AIDS communication in South Africa be anchored on the cultural norms and beliefs of the target communities. Important aspects of the indigenous language such as proverbs and idioms should be exploited to maximise success at behaviour change.

Second, the designers of any intervention should as a matter of urgency start with needs assessment (Madelief, Schaalma, Bartholomew & Van Den Boerne 2008). Needs assessment involves designers of the intervention going into the target communities to assess their priority needs and experiences and use these as basis for the design of their intervention instrument. According to Madelief *et al.* (2008:14–15), such assessments will include epidemiological analyses of behavioural and environmental causes of health problem as well as the sociological analyses of the resources or capacity of the community. They contend that the primary goals of a needs assessment include getting a full understanding of priority population's problems, its character and its strength. The need to reevaluate theatre's intervention methodology has become crucial given the rising prevalence of HIV/AIDs across the country.
